# Oral Immunotherapy With Extensively Hydrolyzed Milk for a 12-Year-Old Child With Persistent, Severe Cow’s Milk Allergy

**DOI:** 10.7759/cureus.59188

**Published:** 2024-04-28

**Authors:** Kenta Horimukai, Misako Kinoshita, Noriko Takahata

**Affiliations:** 1 Department of Pediatrics, Jikei University Katsushika Medical Center, Tokyo, JPN

**Keywords:** milk hypersensitivity, infant formula, immunotherapy, food hypersensitivity, anaphylaxis

## Abstract

A 12-year-old girl with severe cow's milk allergy (CMA) was able to safely consume 300 mL of unhydrolyzed cow's milk after three and a half years of oral immunotherapy (OIT) with extensively hydrolyzed milk. The treatment consisted of gradually increasing the intake of hydrolyzed and partially hydrolyzed milk and reintroducing cow's milk. Despite some allergic reactions during treatment, the patient was able to consume more than 200 ml of milk consistently for more than six months without recurrence of symptoms. This case suggests the possibility of an alternative treatment for persistent CMA: not only OIT with cow's milk alone but also a safer introduction to treatment with extensively hydrolyzed formulas.

## Introduction

Cow's milk allergy (CMA) is a leading food allergy in the developed world, with a prevalence of 0.5%-3% at one year of age [1]. Although many infants with CMA develop tolerance with age, cow's milk remains the leading food allergen associated with fatal anaphylaxis in children younger than 16 years in the United Kingdom [2]. In some cases, CMA persists into adolescence, which suggests a persistent anaphylactic risk, highlighting the need for safer oral immunotherapy (OIT) strategies.

For children with high-risk severe CMA, whose symptoms were triggered by small amounts of cow's milk consumption up to 12 years of age and who had undergone complete elimination, we attempted to introduce safer OIT induction using extensively and partially hydrolyzed formulas. The extensively hydrolyzed formulas used were MA-1® and MA-mi®, while Eakachan® was a partially hydrolyzed formula, all products of Morinaga Milk Industry Co., Ltd., Tokyo, Japan.

## Case presentation

We present the case of a 12-year-old girl with severe CMA who, after three and a half years, was able to safely consume 300 ml of cow's milk through the gradual introduction of extensively hydrolyzed formulas (MA-1^®^ and MA-mi^®^), a partially hydrolyzed formula (Eakachan^®^), and unhydrolyzed cow's milk. This work was approved by the Jikei University School of Medicine Ethics Review Committee (Approval No. 35-446: 12086). Informed consent was obtained from the patient and her guardian. The patient experienced an immediate allergic reaction to cow's milk after consuming yogurt at six months of age, necessitating milk elimination. At her initial presentation, she was highly sensitive to several food allergens, particularly milk (Table 1).

**Table 1:** Characteristics of initial food allergy test results in a 12-year-old girl RIs: reference intervals RIs for total IgE levels were obtained from Martins et al., 2014 [3]. RIs for specific IgE antibody titers were defined as the range within which they are typically negative.

Total IgE (RIs: 2–696 IU/mL)		956
Specific IgE (RIs: < 0.35 kUA/L)	Milk	39.5
	Casein	46.42
	β-lactoglobulin	3.27
	Egg white	23.5
	Ovomucoid	21.9
	Wheat	10.49
	ω5-gliadin	0.23

Three months before presenting to our clinic, she developed anaphylaxis characterized by respiratory symptoms during an oral challenge with 2 mL of cow's milk. Following our initial consultation, she successfully completed an oral challenge with 100 mL of extensively hydrolyzed milk MA-1^®^ and maintained this intake at home for three months. We then introduced 10 mL of extensively hydrolyzed milk MA-mi^®^ into MA-1^®^, gradually increasing MA-mi^®^ to 100 mL. She tolerated 100 mL of MA-mi^®^ without adverse reactions after one month. Treatment progressed to incorporating 5 mL of partially hydrolyzed milk Eakachan^®^ with MA-mi^®^, eventually increasing to 100 mL. Two months later, she could consume 100 mL of Eakachan^®^ without allergic symptoms.

After a negative result in an oral challenge combining 0.1 mL of milk with 100 mL of Eakachan^®^, the milk quantity was gradually increased. Two months into the challenge, the dose was cautiously increased owing to mild pruritus noted after the consumption of 1.2 mL of milk. After five months, the quantity was increased to 5 mL, and after seven months, to 20 mL. Eight months after milk introduction into the diet, symptoms such as nasal discharge, sneezing, hives, and dyspnea were observed after consumption of 100 mL of Eakachan^®^ and 20 mL of milk, along with sleep deprivation due to exam preparation. The symptoms were relieved after loratadine (10 mg) administration and adrenaline injection.

**Figure 1:**
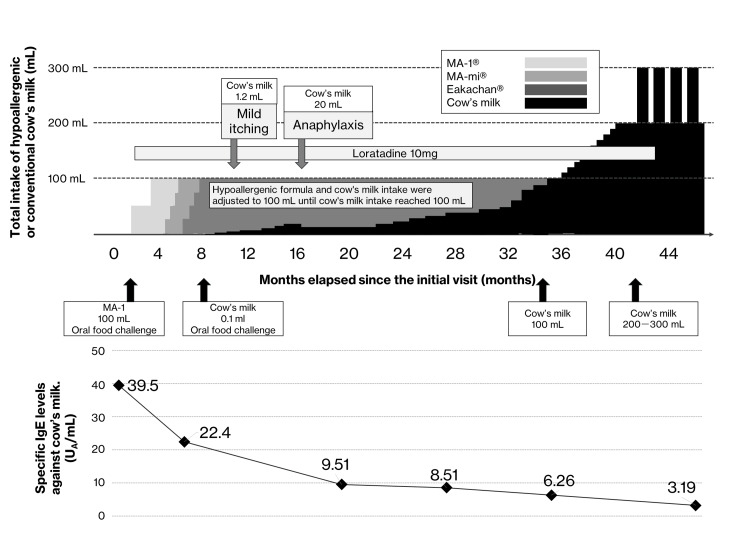
Combined intake of hypoallergenic cow's milk: clinical events and therapeutic interventions throughout the treatment course

After 14 months of daily consumption of 100 mL of Eakachan^®^ and 15 mL of cow's milk without allergic reactions, the dose was cautiously increased. After an anaphylactic episode, no allergic reactions were observed with a gradual increase to 100, 150, and 200 mL at 28, 31, and 33 months, respectively. At 34 months, she tolerated 250-300 mL without adverse effects. Furthermore, allergic symptoms did not recur after she consistently consumed more than 200 mL of milk for more than six months. The case course is shown in Figure 1.

Although the patient chose not to undergo confirmation of sustained unresponsiveness and no subsequent oral challenge was performed after milk discontinuation, the milk-specific IgE antibody titer decreased over the treatment course. Furthermore, the previous physician had advised the patient to eliminate all chicken eggs and wheat during the initial consultation. However, through oral food challenges and immunotherapy, the patient reintroduced these foods daily after 14 and eight months, respectively.

## Discussion

The gold standard for managing food allergy is accurate diagnosis and elimination of allergenic foods. However, elimination alone does not ensure patient safety. In a study of 88 children with CMA who were advised to avoid certain foods, 35 (40%) experienced allergic reactions owing to food mishandling over a one-year period [4]. In addition, research on children with food-induced anaphylaxis in pediatric emergency departments has shown that older children have more severe allergic reactions compared with toddlers [5].

Another issue is that continued milk elimination does not always result in remission. Kubota et al. observed that spontaneous remission by 12 years of age was unlikely when three factors were present: a high milk-specific IgE titer at six years of age (cut-off: 12.7 kUA/L), a history of anaphylaxis due to milk, and strict avoidance of cow's milk [6]. The conditions in our case were consistent with these, indicating that spontaneous remission was not expected.

In a recent study, 60 infants younger than six months with CMA were randomized to receive a non-allergenic amino acid-based or less allergenic extensively hydrolyzed formula. Remission rates were compared at 12 months [7]. Three percent and 48% of infants in the amino acid-based and extensively hydrolyzed formula groups, respectively, achieved remission, with no severe allergic symptoms associated with the extensively hydrolyzed formula.

MA-1^®^, MA-mi^®^, and Eakachan^®^ are hypoallergenic formulas commonly used in Japan for children with cow's milk allergy. MA-1^®^ and MA-mi^®^ are extensively hydrolyzed and have low allergenicity. However, based on lymphocyte stimulation test results and additional research, MA-mi^®^ is suspected to be more allergenic than MA-1^®^ [8].

Both MA-1^®^ and MA-mi^®^ are enzymatically hydrolyzed and ultrafiltered to reduce the allergenicity of their casein and whey proteins. However, unlike MA-mi^®^, MA-1^®^ does not contain whey protein and the molecular weight distribution differs between the two products. Specifically, the maximum molecular weight of MA-1^®^ is approximately 1000 Da, whereas the maximum molecular weight of MA-mi^®^ is approximately 2000 Da, which may explain why MA-mi^®^ has a higher allergenicity [8]. Additionally, Eakachan^®^ is a partially hydrolyzed formula and has a significantly greater potential to induce allergenicity and oral immune tolerance than MA-mi^®^ [9]. This difference in allergenicity can be attributed to the fact that Eakachan^®^, which is only partially hydrolyzed, contains 2.9% of molecules above 3500 Da, whereas MA-mi^®^ contains virtually no high molecular weight molecules [9].

Accordingly, we administered these formulas to the current case in a staged approach. Nevertheless, during a sleep deprivation period, the patient experienced anaphylaxis after ingesting 100 mL of Eakachan^®^ and 20 mL of cow's milk, necessitating an adrenaline injection. Research in adults with peanut allergy has shown that the allergen tolerance threshold is 45% lower under sleep deprivation [10]. Given the myriad life events that adolescents face, OIT in high-risk populations such as this patient requires careful consideration.

This work has limitations, such as being a single case report and the ambiguity of whether tolerance developed spontaneously. In addition, milk-specific IgE antibody titers tended to decrease in affected children following the introduction of a hypoallergenic formula for immune tolerance induction. After successful induction of remission, immune tolerance induction by cow's milk alone was considered; however, Eakachan^®^, a partially hydrolyzed formula, may also contribute to immune tolerance induction [9]. In other words, consumption of Eakachan^®^ may have an additive effect beyond that of cow's milk alone in inducing immune tolerance. Furthermore, it has been reported that infants with cow's milk allergy show improved height and weight gain after consumption of extensively hydrolyzed formulas [11]. Therefore, potential reasons for continuing Eakachan^®^ alongside the introduction of cow's milk include not only the induction of immune tolerance but also the additional nutritional benefits.

Furthermore, the patient underwent prolonged treatment (for three and a half years). However, compared with another study in which children with severe CMA received OIT with 3 mL of milk daily, with 27%, 52%, and 61% tolerating 25 mL at one, two, and three years, respectively [12], three years of treatment may be warranted for persistent CMA up to 12 years of age. This case provides insight into the evolving field of OIT for CMA and suggests a potential alternative treatment.

## Conclusions

The results of this case report suggest that OIT with extensively and partially hydrolyzed milk formulas may be an effective strategy for the treatment of persistent CMA. Over three and a half years, the patient achieved a significant increase in milk tolerance and successfully consumed up to 300 mL without adverse reactions. These results highlight the potential of OIT protocols to improve food allergen tolerance.
